# Dental Quacks

**Published:** 1872-12

**Authors:** G. O. Howard

**Affiliations:** Galena.


### ARTICLE II.

### "*Dental Quacks.*"

BY DR. G. O. HOWARD, OF GALENA.

Read before the Illinois State Dental Society.

By the medical profession it is considered quackery to practice medicine without first taking a thorough course of study, and obtaining a diploma from some respectable medical college. The dental profession is not yet so strict with its members, while it is desirous that all should be graduates of some respectable dental college, yet it is admitted that there are good and honorable dentists who do not hold a diploma. Our profession is too young yet to draw the lines as closely as does the medical profession in this respect, but we have not to look far into the future to see the time when all men that practice dentistry, and are not graduates, will be branded as quacks by the profession. Let us then, who do not possess a thorough dental education, nor hold a diploma, hasten our efforts to obtain them. Dentistry is upon the forward march, and that so rapidly, that we must use everything within our reach to keep up with it, and I assure you, from personal experience, that without a thorough college education, it is up-hill work to keep apace with our profession.

A quack means a boaster, an ignorant practitioner, an empiric; these definitions cover more ground in our profession than we at first may think. It would be tedious were I to attempt a description of each kind of quacks and how they perform, so I will confine myself to three or four of the worst types, who they are, and what, perhaps, is the best manner in which to treat them.

Let us look first to the class of so-called dentists that are self-graduates. They are quite numerous and dangerous. By a self-graduate, I mean one who withdrew from his preceptor's office after a studentship of a few months, fully confident of his ability to perform all dental operations. He has read somebody's treatise upon dentistry, and has seen his preceptor perform most all ordinary operations, which he knows he can do just as well; to be sure he has never tried, but they are so simple that anybody could do them after once seeing. He collects together a few instruments; it does not matter much what they are; he can get along with almost anything; next he gets a few loud circulars, something after this style:—"Dr.———, from———, will be at———on the——day of———, where he will be prepared to practice dentistry in all its branches, in the latest and most improved style of the art, with prices within the reach of all, and all operations warranted to give satisfaction or the money refunded." Perhaps the Dr. treats himself to a puff, in which he hints very loudly about his long experience and abilities, &c., &c. Fortified in this manner, he makes his appearance upon the state as a practising dentist in some small town. No doubt all of us here know of one or more of this class, so it will not be necessary for me to detail the result of his operations, more than to say that he is dangerous to the prosperity of our profession, because all, or nearly all who patronize him, lose their confidence in the durability of dental operations. How often do we hear it said: "I do not believe in filling teeth, for Dr.—— filled some for one of my friends, and the fillings have all come out, and Dr.—— was called a good dentist," or other remarks similar to this?

We may divide this type into two classes; one is after practice, that he may improve himself, and at the same time make a living. The other is a tooth stuffing expedition, and cares only for the dollar. One tries every time to do better than he did the last; the other tries to get the dollar into his pocket quicker than he did before. One never misses an opportunity to gain useful information; the other you cannot give any instruction to; he already knows it all. One works with honest intentions; the other works dishonestly. For the prosperity of our profession and the welfare of our patrons, what is the best manner to treat these two classes of self-graduates? They are both quacks, and it appears to be professional to discourage quackery, yet one has honest intentions, and these should have their reward. We know that he is pursuing a course to obtain knowledge, at the expense of an unsuspecting public, and giving them worse than nothing in return; he does not look at it as we do. Here is honesty and genius upon the wrong course; let us extend a helping hand and try to turn him in the right direction, and we may be rewarded sometime by seeing him with his shoulder to the wheel doing better work than we ever did. The other we would not hesitate to discountenance at every opportunity. He "has no rights that we are bound to respect."

There is another type of quacks that, perhaps, are as numerous as the self-graduates, and far more dangerous and contemptible. This is a class that every honest dentist looks upon as a disgrace to the profession; they are men in years and in appearance, and that is the limit of their manhood. This class has many grades in it, and I will occupy your time only to describe one of the best grades. In the first place, he is a smiling, smooth-talking, smirking hypocrite; he does not stop in one place more than two or three years, and his introduction at every place is a card from his predecessor, informing the public that he has sold out to Dr.——, and cheerfully recommends him to them as a first-class dentist. This is followed in a few days by a grand puff in the local newspaper, of the refurnished office, new instruments, latest improvements, laughing gas, teeth extracted without pain, splendid plate work on exhibition, &c., &c., until the public are almost led to believe that the town has received a valuable acquisition in the new dentist. He attends the most popular church and takes a lively interest in its welfare, and as soon as permitted, becomes a member. Such an act would not be criticised here, if we did not know him to be a hypocrite. Next, he circumvents the M. D's. of the place, doing their dentistry for nothing or at a discount, also that of the ministers. He joins all the secret societies that he can force himself into. Goes calling at every opportunity, attends all parties, and in fact, never fails to avail himself of every opportunity to make himself popular. He is very affable to all. Now this looks all right to the public, but to us who know him, it is disgusting. He advertises largely and he is in a short time overrun with work; his office is always full, and if we were to step in his neighbor's office, (who, by the way, is one of those honest, hard studying, progressive dentists that will not stoop to hypocrisy,) more than likely you will find him alone, with some book, or at his work-bench trying to work out some mechanical problem; he will tell you that business is dull. Thus far Dr. Quack is a success, but it is not long before his real self begins to show; he cannot sustain his assumed character; he must stoop to mean dirty tricks; one thing in particular, he never fails to run down his neighbor's operations, and no matter whether they are good or bad, he removes all that he is permitted to, and some without permission. Everything he buys is on credit, and his creditors, in order to get their pay, must go to him to get their dentistry done. He is directly opposed to paying out money if he can help it. He never attends any dental convention, he cannot afford it; but if he is upon speaking terms with his neighbor who does, he will be sure to drop in and find out everything new, under the pretense of a friendly call; in this way he saves the time and expense of attending the convention; perhaps he may be upon very friendly terms with you face to face, and injure you at every chance behind your back. You may establish a list of prices with him and he will undercut you at every chance There is no dependence to be placed upon him; you cannot tell where to find him. As a mechanical dentist he is poor; as an operator, worse. I have known men of this class to combine dentistry with the barber trade; and to travel the country with a stylish horse and sulky, with a box of tools strapped up under the seat, distributing two kinds of circulars, one telling what the man is and what he can do, and the other telling what the horse is and his pedigree.

All this is true, and no doubt we all know of such men, and have been troubled to know what to do to get rid of them. I have seen so many of this kind that I am sometimes almost ashamed to tell strangers that I practice dentistry for a living. Our laws protect them, or perhaps they might receive a just punishment from the hands of those they injure. It will not do to tell the public what they are, because if we do we lay ourselves liable to the accusation of running them down for selfish purposes. It will not do to advertise and play the hypocrite as they do, in order to keep up your practice, because that is not honorable. What then is to be done? I can think of no way to succeed in cleaning them out, except to have nothing to do with them. Practice honesty when consulted about Dr. Quack's operations, give an honest report, and do not hesitate to renew them if it be necessary and if it is desired by the patient. I mean do not have any professional delicacy about it.

We, of the present generation in this State, will never be entirely clear of them, even if the bill that is now before the Legislature should become a law, as it does not affect this class of men that are now in practice.

There is another class that are quacks. They are men that have received a good dental education, and when they first started in practice, and perhaps for some years after, were sound in their action, and associated with other dentists in a friendly manner, receiving and imparting useful knowledge by so doing, but they have gradually got the "big head," and have discontinued their intercourse with results very much to their detriment. We find them plodding along in the same old tracks of years ago, all puffed up by their imagined superiority over others, thinking they know more than the whole dental profession put together, and telling their patients so at every opportunity. They can perform a fair operation and make a good piece of plate work. It is very disagreeable to practice in the same place with such a man, but what will you do about it? I would say have nothing to do with him, for if you handle dirt you will soil your hands.

Thus far I have talked about those who have not a thorough college education, and perhaps some here who are graduates think that if every man that practises dentistry were a graduate, we would not be troubled by these. For the benefit of those who think so, I will read a copy of an advertisement that I clipped from a newspaper, published in the northern part of this State:

"Dr. ——, Dentist.

"Those wishing first-class dental operations should visit Dr.——, graduate of the —— Dental College; he has had years of practice, and will guarantee entire satisfaction at reasonable charges."

And again, from a graduate of a Dental College at Philadelphia, a poetical sort of a chap, who advertises in this manner:

"Would shield defenseless infancy from harm,
Call on B. & S., Dentists.
And patient merit never wants a friend,
Call on B. & S., Dentists.
But when from loss of teeth the food must pass,
A crude, one rigid, one unbroken mass,
To the digestive organs, who can know
What various forms of complicated woe
May rise terrific from that single source, breeds of fearful things.
Call on B. & S., Dentists.
The teeth, deciduous, tartar and decay.
Call on B. & S., Dentists.
In secret whisper the kind untruth impart,
There is a remedy—the dental art
Can every varying tone with ease restore,
And give the music sweeter than before.
Call on B. & S., Dentists."

This was published in the Galena *Daily Gazette*, and by a graduate of the Pennsylvania College, I think. Here is some more from a College graduate, in the local of a paper published in a Western City—a paid puff.

"*New Dental Rooms.*—We yesterday visited the newly opened Dental Rooms of ———. We were no less astonished than pleased at the information which the visit afforded us. The reception, or operating room, is elegantly fitted up with all the appliances known to modern dentistry. On one side of the room is an elegant case filled with drawers, in which are kept a multitudinous assortment of every instrument used for the extraction of every kind of teeth in all stages of decomposition. Here, likewise, are kept the different kinds of articles used for filling teeth, such as gold leaf, metal and other durable articles. Adjoining this case is his prescription department, in which he keeps all the different kinds of medicines used in alleviating the pain so incidental to pulling or filling teeth. On the east side of the room is a well filled library of the best standard and special works published on dentistry; adjacent are illustrated dental charts, showing the formation of teeth in the jaws, and their relation to the remainder of the head and body. A very neat operating chair is conveniently situated. A rare cabinet of stuffed birds, pictures, etc., occupy a portion of the walls, and other places in the rooms. New carpets are laid on the floor. The laboratory and mechanical room is adjacent, and is supplied with all the latest improvements in the way of dental machinery. The doctor administers nitrous oxide or laughing gas, to those who prefer to remain unconscious or painless while they are being operated on. Dr.—— is a highly cultivated and experienced dentist, takes a just pride in his profession, and has come among us for the purpose of a permanent residence. Being a gentleman of means, he proposes investing his capital, and becoming an additional recruit to the rapidly augmenting army that have an all abiding faith in the present and future growth of our city."

Now, will you be surprised when I tell you that this man of means left that city in less than one year, in debt to his boarding mistress, and owed the editor for his advertising? You will see there are black sheep in all flocks. Is there no way that we can rid the country of these imposters? I think there is one, and that is to educate the public to discriminate between the imposter and the true man, and there is no better place to commence than here. Let us do as our St. Louis brothers have done, publish a magazine, something larger and and more attractive than their's, or better, perhaps, aid our St. Louis brothers to enlarge the work they have commenced.

Thus far, perhaps, we have been talking about others outside of this Society; now let us look at home, and see if we are not all quacks to some extent; let us all ask ourselves this question—do we ever undertake to treat cases that we do not understand? Do we not have cases that we feel perfectly confident we understand, and find afterwards that we know nothing about them? Are we not ignorant practitioners and experimenters? Are we anything but boasters when we say, upon examining others' operations, that if we had performed them they would have been done better? Do we find ourselves saying anything about our neighbor that would have been a little better not said?

These things, if practised, brand us as quacks. Let us examine ourselves closely; there are none of us so perfect that we need not criticize our own actions, once in a while at least.

